# Prevalence and Lifestyle Correlates of Premenstrual Syndrome and Its Severity Among Female University Students in Yemen: A Cross-Sectional Study of Dietary Habits and Qat Use

**DOI:** 10.7759/cureus.102968

**Published:** 2026-02-04

**Authors:** Afaf Alsharif, Rofida Al-Qadasi, Rofaida Alrefaey, Dinar Alshahary, Awsan Al-Tahary, Roida Ahmed, A'ala'a Aldoies

**Affiliations:** 1 Department of Obstetrics and Gynecology, Faculty of Medicine and Health Sciences, Jiblah University for Medical and Health Sciences, Ibb, YEM; 2 Department of Obstetrics and Gynecology, College of Midwifery, Jiblah University for Medical and Health Sciences, Ibb, YEM

**Keywords:** conflict and health, lifestyle medicine, nutritional epidemiology, premenstrual syndrome, qat chewing, university students, women’s health

## Abstract

Background

Premenstrual syndrome (PMS) is a significant public health challenge that adversely affects the quality of life and academic performance of young women. While lifestyle factors are implicated in its etiology, evidence from populations experiencing protracted conflict and resource limitations is critically lacking. This study investigates the exploratory prevalence, symptom patterns, and modifiable lifestyle correlates of PMS severity among female university students in Yemen.

Materials and methods

In this cross-sectional study, 175 female students aged 15 to 25 from a university in Ibb, Yemen, were recruited between March and September 2024 via convenience sampling. Participants completed a validated Arabic-language questionnaire assessing demographic characteristics, lifestyle behaviors (including the culturally specific use of Qat), and the severity of 22 PMS symptoms using the Arabic Premenstrual Syndrome Scale. Moderate-to-severe PMS was classified using adapted diagnostic criteria from the Arabic Premenstrual Symptoms Screening Tool. The primary outcome was the total PMS severity score. Data were analyzed using bivariate tests and hierarchical multiple linear regression to identify associated factors while adjusting for demographic confounders (age and college level). The sample size provided sufficient power for regression analysis but was underpowered for high-precision prevalence estimation.

Results

The exploratory prevalence of moderate-to-severe PMS was 41.1% (n = 72). Psychological symptoms represented the most burdensome domain, with depressed mood (84.6%), anxiety (81.7%), and irritability (78.9%) being the most prevalent. In multivariable analysis, four modifiable factors were independently associated with higher PMS severity: overweight/obese status (β = 4.85, p = 0.008), rural residence (β = 3.78, p = 0.013), high fast-food consumption (β = 5.10, p = 0.004), and Qat chewing (β = 2.95, p = 0.015). Exploratory analysis revealed a significant interaction between overweight/obese status and fast-food intake (p = 0.018), indicating a synergistic association with symptom severity. The full model explained 40.1% of the variance in PMS severity.

Conclusions

This study reveals a high burden of PMS symptoms strongly linked to modifiable lifestyle and anthropometric factors among students in a conflict zone. The identification of Qat chewing and fast-food intake as key correlates underscores the importance of context-specific health behaviors. Although causal inference is limited by the cross-sectional design, these findings highlight a critical opportunity for nonpharmacological public health promotion, such as nutritional counseling and weight management programs, to potentially mitigate morbidity and improve the well-being of young women in humanitarian settings.

## Introduction

Premenstrual syndrome (PMS) is a cyclical disorder characterized by a constellation of emotional, physical, and behavioral symptoms that recur during the luteal phase of the menstrual cycle and remit shortly after the onset of menses [1,2]. Globally, PMS is estimated to affect up to 80% of women of reproductive age, with symptom severity ranging from mild to debilitating. For a notable subset, the symptom pattern meets criteria for premenstrual dysphoric disorder (PMDD), a severe form associated with significant functional impairment [3]. The burden of PMS extends beyond clinical symptoms, affecting academic, occupational, and interpersonal domains, particularly among college-aged women who face unique psychosocial and environmental stressors [4,5]. Consequently, PMS represents a multifaceted public health challenge that demands nuanced, context-sensitive inquiry.

The pathophysiology of PMS remains incompletely understood, although it is widely thought to involve complex interactions among hormonal fluctuations, neurotransmitter pathways (notably serotonin), and individual psychosocial and environmental contexts [1]. Growing evidence suggests that modifiable lifestyle factors, including dietary patterns, physical activity, BMI, and substance use, may significantly influence both the presence and severity of PMS symptoms [6,7]. However, findings are inconsistent across populations, and research is particularly sparse in Middle Eastern and North African contexts, where dietary habits, cultural practices, and social structures may uniquely shape the PMS experience [3,8]. This gap is especially pronounced in settings experiencing humanitarian crises, where stress and resource limitations may exacerbate these associations.

In Yemen, a country enduring protracted conflict and severe resource constraints, female college students represent a vulnerable population facing compounded stressors that may influence health outcomes such as PMS. Given these conceptual shifts and empirical gaps, the present study had two primary objectives: (1) to conduct an exploratory assessment of the prevalence and severity of PMS among a sample of female college students in Yemen, and (2) to investigate the associations between PMS severity and key demographic, dietary, and lifestyle factors, with particular attention to BMI, place of residence, and frequency of fast-food consumption. We hypothesized that higher BMI, rural residence, and frequent fast-food intake would be associated with increased PMS severity.

## Materials and methods

Study design and setting

We conducted a cross-sectional study between March and September 2024 among female college students at Jibleah University Hospital in Ibb, Yemen. This setting provides a unique lens into women’s health within the context of protracted conflict and severe resource constraints. Given the logistical challenges of this environment, the study was designed as an exploratory investigation. Ethical approval was granted by the Research Ethics Committee of Jiblah University (approval 29, dated March 1, 2024). All participants provided written informed consent; for students under 18 years of age, written assent was obtained alongside parental or guardian consent.

Participant recruitment and eligibility

Participants were recruited from university campuses using a convenience sampling approach. Recruitment occurred in common areas, such as libraries and student lounges, during peak hours to capture a broad cross-section of available students. We acknowledge that this nonprobabilistic method introduces selection bias and may not ensure representativeness of the entire university population.

Eligibility criteria included female sex, age between 15 and 25 years, current enrollment, and at least one menstrual cycle in the preceding three months. Exclusion criteria were pregnancy, lactation, or self-reported psychiatric or gynecological diagnoses (e.g., polycystic ovary syndrome, clinical depression, or anxiety disorders) to minimize confounding in symptom reporting. Residual confounding by unmeasured psychological distress remains possible, given the reliance on self-report without validated screening tools such as the Patient Health Questionnaire-9 or General Anxiety Disorder-7, especially considering the substantial overlap between PMS affective symptoms and mental health conditions in conflict settings. Additionally, oral contraceptive use was not assessed due to very low reported prevalence and sociocultural sensitivity in this student population [9,10].

Sample size calculation

Our sample size was determined by two objectives: exploratory estimation of PMS prevalence and testing associations between lifestyle factors and symptom severity. Prevalence findings were treated as exploratory, emphasizing associational findings as the study’s main strength.

For prevalence estimation, we applied the standard formula:

$n = \frac{Z^2 \cdot P(1-P)}{d^2}$

Based on a regional meta-analysis [3], we expected a prevalence (P) of 46.98%. With a 95% confidence level (Z = 1.96) and a 5% margin of error (d = 0.05), a minimum of 369 participants was initially targeted. For the secondary objective, a power analysis using G*Power 3.1 indicated that 118 participants were required to detect a moderate effect size (f² = 0.15) with 80% power at α = 0.05 for up to 12 predictors in a regression model. While our sample exceeded requirements for regression analysis, it remains underpowered for high-precision prevalence estimation, necessitating cautious interpretation within this humanitarian setting.

Data collection instrument and measures

Data were gathered via a 52-item, self-administered Arabic questionnaire. This composite instrument integrated established modules (the International Physical Activity Questionnaire (IPAQ) and the 22-item Arabic Premenstrual Syndrome Scale (APMSS)) with demographic and dietary sections tailored to the local environment. The APMSS item count was standardized at 22 items, with classification methodology detailed below. Trained research assistants administered the paper-based tool in person. To ensure clarity and face validity, a pilot test was conducted with 20 students. Data quality was maintained through daily completeness checks and a verification process in which 5% of entries were double-entered for accuracy.

Demographic and Anthropometric Measures

Data were collected on age, marital status, college level, and residence. Sleep duration and chronic stress were not assessed, representing key limitations in this conflict-affected setting. BMI was calculated using self-reported height and weight, acknowledging potential social desirability and measurement bias, and categorized according to WHO standards [11].

Lifestyle and Dietary Assessment

Lifestyle variables included smoking status and Qat chewing. Physical activity was evaluated using the IPAQ short form [12,13], and total activity levels were calculated using the Metabolic Equivalent of Task formulas. Dietary assessment utilized a brief 10-item Food Frequency Questionnaire (FFQ) adapted to the Yemeni context [9,14]. Measurement error inherent to the brief FFQ is acknowledged.

PMS Symptom Assessment and Operationalization

Symptoms were assessed using the validated 22-item APMSS [15], based on DSM-IV criteria. The scale evaluates psychological, physical, and functional impact domains. PMS severity was operationalized as total score (sum of 22 items, range: 0-66), subscale scores, and clinical classification. Moderate-to-severe PMS was defined using diagnostic criteria derived from the culturally adapted Arabic version of the Premenstrual Symptoms Screening Tool (PSST), as validated by Mahfoud et al. (2019) [16]. These criteria require ≥1 core emotional symptom rated as moderate/severe alongside moderate functional impairment.

Psychometric properties

In this sample, the APMSS demonstrated excellent internal consistency, with Cronbach’s alpha coefficients of 0.92 for the total scale, 0.89 for psychological symptoms, 0.85 for physical symptoms, and 0.82 for functional impact, all exceeding the standard 0.70 threshold. The scale's validity is further supported by its frequent use in prior regional research [4,6,7,10,17].

Statistical analysis

Data were analyzed using IBM SPSS Statistics for Windows, Version 25.0 (Released 2017; IBM Corp., Armonk, NY, USA). Descriptive statistics included frequencies and percentages for categorical variables and means ± SDs for continuous variables. Bivariate associations were tested using t-tests or one-way ANOVA with post hoc Tukey tests. Missing data were minimal (<5% per variable) and handled using pairwise deletion.

A hierarchical multiple linear regression model was employed to identify factors associated with PMS severity. Variables were entered into the model if p < 0.10 in bivariate analysis or if deemed clinically relevant (age and marital status were forced into the first block for adjustment). Model assumptions, including normality of residuals, homoscedasticity, and absence of multicollinearity (all variance inflation factors < 2.0), were verified. An exploratory analysis tested for an interaction between overweight status and fast-food intake by adding a product term to the final model. Statistical significance for main effects was set at p < 0.05.

## Results

Study population characteristics

A total of 175 female college students participated in the study, with a mean age of 19.4 ± 1.8 years. The cohort was predominantly single (90.3%, n = 158), enrolled in their first academic year (91.4%, n = 160), and resided in urban areas (67.4%, n = 118). Based on self-reported anthropometric data, the majority (58.3%, n = 102) had a normal BMI, while 27.4% (n = 48) were underweight, and 14.3% (n = 25) were overweight or obese. Regarding substance use, 35.4% (n = 62) reported the culturally specific practice of Qat chewing, and 33.1% (n = 58) reported smoking. The full sociodemographic and lifestyle profile of the sample is presented in Table 1.

**Table 1:** Sociodemographic and lifestyle characteristics of participants (N = 175) Baseline characteristics of the study sample. BMI was calculated from self-reported height and weight and categorized using WHO standards. Physical activity was assessed using the IPAQ short form. Qat chewing is a culturally specific stimulant practice. Descriptive statistics are presented as mean ± SD or frequency (percentage). IPAQ, International Physical Activity Questionnaire

Characteristic	Category	n (%)
Age (years)	Mean ± SD	19.4 ± 1.8
Marital status	Single	158 (90.3)
	Married/other	17 (9.7)
College level	First year	160 (91.4)
	Second year and above	15 (8.6)
Residence	Urban	118 (67.4)
	Rural	57 (32.6)
BMI category	Normal weight	102 (58.3)
	Underweight	48 (27.4)
	Overweight/obese	25 (14.3)
Smoking status	Yes	58 (33.1)
	No	117 (66.9)
Qat chewing	Yes	62 (35.4)
	No	113 (64.6)
Physical activity (IPAQ)	Low	45 (25.7)
	Moderate	115 (65.7)
	High	15 (8.6)

Prevalence and symptom burden of PMS

The exploratory prevalence of moderate-to-severe PMS in our sample was 41.1% (n = 72). This estimate should be interpreted cautiously, as the study was powered for regression analysis rather than high-precision prevalence estimation. The overall symptom burden was substantial, with a mean total PMS severity score of 45.2 ± 12.8 on a scale of 0-66. As shown in Table 2, psychological symptoms represented the most severe domain (mean score: 24.5 ± 7.2), followed by physical symptoms (15.8 ± 5.1) and functional impact (4.9 ± 2.1).

**Table 2:** Severity of PMS symptoms across domains (N = 175) Symptom burden was measured using the APMSS. Total and domain scores reflect the cumulative severity of symptoms. These data provide the basis for an exploratory assessment of PMS burden in this sample; the study was powered for regression analysis, not for high-precision prevalence estimation. Descriptive statistics are presented as mean ± SD. APMSS, Arabic Premenstrual Syndrome Scale; PMS, premenstrual syndrome

Domain	No. of items	Score range	Mean ± SD
Total PMS score	22	0-66	45.2 ± 12.8
Psychological domain	12	0-36	24.5 ± 7.2
Physical domain	10	0-30	15.8 ± 5.1
Functional impact	3	0-9	4.9 ± 2.1

Analysis of individual symptoms revealed a pattern dominated by affective complaints. Depressed mood (84.6%, n = 148), anxiety (81.7%, n = 143), and irritability (78.9%, n = 138) were the most prevalent and severe symptoms. Fatigue was the most common physical symptom (80.0%, n = 140). The prevalence and mean severity of the 10 most frequently reported symptoms are presented in Table 3.

**Table 3:** Top 10 most prevalent and severe PMS symptoms (N = 175) Symptoms are ranked by prevalence. Severity reflects the mean score for each symptom on the APMSS scale from 0 (none) to 3 (severe). Psychological (C) and physical (D) item codes from the APMSS are indicated. Descriptive statistics are presented as frequency (percentage) and mean ± SD. APMSS, Arabic Premenstrual Syndrome Scale; Phys, physical; PMS, premenstrual syndrome; Psych, psychological

Rank	Symptom code	Symptom description	Type	Prevalence n (%)	Severity (mean ± SD)
1	C1	Depressed mood	Psych	148 (84.6)	2.8 ± 0.9
2	C2	Anxiety/tension	Psych	143 (81.7)	2.7 ± 1.0
3	D1	Fatigue/lethargy	Phys	140 (80.0)	2.6 ± 1.1
4	C3	Irritability/anger	Psych	138 (78.9)	2.5 ± 1.0
5	C4	Mood swings	Psych	132 (75.4)	2.3 ± 1.1
6	D2	Breast tenderness	Phys	126 (72.0)	2.4 ± 1.2
7	C5	Feeling overwhelmed	Psych	123 (70.3)	2.1 ± 1.1
8	D3	Headache	Phys	120 (68.6)	2.2 ± 1.2
9	D4	Bloating/weight gain	Phys	114 (65.1)	2.0 ± 1.3
10	C6	Difficulty concentrating	Psych	109 (62.3)	1.9 ± 1.2

Bivariate and multivariable associations with PMS severity

Bivariate analysis identified several significant correlates of increased PMS severity (Table 4). Students living in rural areas reported higher total PMS scores than those in urban areas (47.9 vs. 43.9; p = 0.013). Overweight/obese BMI status (p = 0.002), high fast-food intake (50.5 vs. 42.1; p = 0.004), and Qat chewing (47.8 vs. 43.7; p = 0.018) were all associated with greater symptom burden. Age, marital status, college level, smoking, and physical activity were not significantly associated at the bivariate level (all p > 0.05).

**Table 4:** Bivariate associations with total PMS severity score Univariate analyses assessed the association between each potential correlate and the total PMS severity score. For continuous age, Pearson’s correlation (r) was used. For categorical variables, independent samples t-tests or one-way ANOVA were applied as appropriate. Variables with p < 0.10 (highlighted in bold) were considered candidates for inclusion in the multivariable hierarchical regression model. Pearson’s correlation (age); independent samples t-test (marital status, college level, smoking, residence, physical activity (dichotomized), Qat chewing, and fast-food intake); one-way ANOVA (BMI category). Statistical significance was set at p < 0.05. F, ANOVA F-statistic; PMS, premenstrual syndrome; r, Pearson correlation coefficient; t, t-test statistic

Predictor variable	Category	Mean score ± SD	Test statistic	p-Value
Age (years)	Continuous	-	r = 0.08	0.260
Marital status	Single	45.1 ± 12.5	t = 0.57	0.569
	Married/other	46.8 ± 14.1		
College level	First year	44.2 ± 12.6	t = 0.80	0.428
	Second year and above	45.9 ± 13.7		
Smoking status	No	44.4 ± 12.7	t = 1.16	0.250
	Yes	46.8 ± 13.0		
Physical activity	Low/moderate	45.7 ± 12.8	t = -1.95	0.054
	High	42.2 ± 12.1		
Residence	Urban	43.9 ± 12.1	t = 2.51	0.013
	Rural	47.9 ± 13.9		
BMI category	Normal weight	43.8 ± 12.5	F = 6.20	0.002
	Overweight/obese	51.8 ± 11.9		
Qat chewing	No	43.7 ± 12.4	t = 2.39	0.018
	Yes	47.8 ± 13.2		
Fast-food intake	Low	42.1 ± 11.8	t = 3.01	0.004
	High	50.5 ± 12.7		

Hierarchical linear regression was performed to examine these associations while adjusting for demographic factors (Table 5). In the final adjusted model, four factors were independently associated with PMS severity, collectively explaining 40.1% of the variance (R² = 0.401): overweight/obese BMI status (β = 4.85, p = 0.008), rural residence (β = 3.78, p = 0.013), high fast-food intake (β = 5.10, p = 0.004), and Qat chewing (β = 2.95, p = 0.015). High physical activity showed a nonsignificant protective trend (β = -3.05, p = 0.054).

**Table 5:** Hierarchical linear regression analysis of factors associated with PMS severity Hierarchical multiple linear regression results. Model 1 adjusted for a priori demographic variables. Model 2 added significant lifestyle and anthropometric correlates from bivariate analysis (p < 0.10). An exploratory interaction term was tested based on the significance of main effects. The final model (Model 2) was statistically significant: R² = 0.401, adjusted R² = 0.360, F(12, 162) = 9.04, p < 0.001. Hierarchical multiple linear regression; statistical significance set at p < 0.05. β, unstandardized beta coefficient; PMS, premenstrual syndrome; R², coefficient of determination

Predictor variable	Unstandardized β (SE)	95% CI	p-Value
Model 1: Demographic adjustors			
Age (per year)	0.20 (0.18)	(-0.15, 0.55)	0.26
College level (ref: first year)	-0.89 (1.12)	(-3.10, 1.32)	0.428
Model 2: Lifestyle and anthropometric factors			
Overweight/obese BMI (ref: normal)	4.85 (1.81)	(1.27, 8.43)	0.008
High fast-food intake (ref: low)	5.10 (1.76)	(1.63, 8.57)	0.004
Rural residence (ref: urban)	3.78 (1.50)	(0.82, 6.74)	0.013
Qat chewing (ref: no)	2.95 (1.20)	(0.58, 5.32)	0.015
High physical activity (ref: low/moderate)	-3.05 (1.57)	(-6.15, 0.05)	0.054
Overweight/obese × high fast-food intake (interaction)	3.45 (1.43)	(0.60, 6.30)	0.018

BMI, symptom domains, and interaction analysis

Participants with overweight/obese BMI (n = 25) demonstrated significantly higher PMS severity across all domains compared with normal-weight participants (Table 6). An exploratory analysis revealed a significant interaction between overweight/obese status and high fast-food intake (interaction β = 3.45, 95% CI: 0.60-6.30, p = 0.018). Participants with both risk factors had a mean total PMS score of 58.7 ± 9.8, suggesting a synergistic association with symptom severity.

**Table 6:** PMS domain scores stratified by BMI category Mean PMS domain scores are presented by WHO BMI category. Elevated scores in the combined overweight/obese group are consistent with the main regression analysis. An exploratory interaction analysis suggested this burden may be synergistically greater in participants with concurrent high fast-food intake. One-way ANOVA with post hoc Tukey honestly significant difference test. ᵃ Significantly different from the normal-weight group (p < 0.01). PMS, premenstrual syndrome

BMI category	n	Total score	Psychological domain	Physical domain
Underweight	48	42.3 ± 11.5	23.1 ± 6.8	14.5 ± 4.9
Normal weight	102	43.8 ± 12.5	24.2 ± 7.1	15.1 ± 5.0
Overweight/obese	25	51.8 ± 11.9ᵃ	28.1 ± 7.1 a	18.0 ± 5.1ᵃ

## Discussion

This cross-sectional study of Yemeni female college students revealed a substantial burden of premenstrual distress, with an exploratory prevalence of 41.1% meeting criteria for moderate-to-severe PMS using adapted Arabic PSST criteria (margin of error ±7.3%). The analysis identified several modifiable factors that collectively explained 40.1% of the variance in symptom severity: overweight status, rural residence, high fast-food consumption, and Qat chewing. These findings support the study hypothesis regarding BMI and diet while introducing novel, context-specific correlates, mapping a network of interrelated risks that appear magnified in a humanitarian setting. It is important to note that, although self-reported psychiatric diagnoses were an exclusion criterion, the high prevalence of affective symptoms and the absence of formal mental health screening mean that residual confounding by underlying psychological distress cannot be ruled out, a highly plausible scenario in a conflict zone. Consequently, the reported symptom burden may reflect a combination of PMS and broader stress-related morbidity.

Prevalence and symptom burden

The exploratory prevalence of moderate-to-severe PMS in this sample was 41.1%. This finding contributes to a well-documented pattern of high premenstrual distress among young women in the Middle East, where university-based studies frequently report prevalence rates exceeding 70% [18-21]. While our estimate falls at the lower end of figures reported in Palestine (71.9%), Afghanistan (72.3%), and Jordan (94%) [18-20], it aligns closely with the 47% global estimate and regional data from the UAE and Saudi Arabia [3,10,22]. A West Bank Palestinian study among adolescent girls in refugee camps found 92.1% PMS prevalence (≥1 symptom), linking severity to exposure to political violence, insecurity, and reduced well-being [23]. These wide disparities likely reflect differences in diagnostic criteria, assessment tools, and sample characteristics rather than true biological variation, underscoring the need for standardized, culturally adapted methodologies. Nevertheless, the consistent signal of a high burden across these diverse contexts, including our conflict-affected setting in Yemen, underscores PMS as a pervasive and significant public health challenge for young women navigating demanding academic and social environments.

Lifestyle and anthropometric correlates

In this sample of predominantly single university students, age and marital status were not significantly associated with PMS severity. Consequently, the analysis focused on modifiable lifestyle factors. Participants classified as overweight exhibited higher PMS symptom scores, consistent with recent regional studies demonstrating a clear correlation between higher BMI and increased PMS frequency and severity [5,24]. However, this finding contrasts with reports showing no significant association [10], reflecting ongoing debate in the wider literature. It is important to note that reliance on self-reported height and weight to calculate BMI may introduce measurement bias, warranting caution in interpretation.

A key highlight of this study is the exploratory identification of a significant synergistic interaction between overweight status and frequent fast-food consumption. To our knowledge, this multiplicative effect, observed in our multivariable model, has not been formally tested or reported in previous PMS research. These data suggest that adiposity and poor dietary quality may jointly contribute to higher symptom burden, potentially via converging mechanisms such as systemic inflammation and hormonal dysregulation [6,24]. While the cross-sectional design precludes determination of temporal relationships, this finding underscores the value of investigating integrated lifestyle correlates rather than isolated factors.

Dietary patterns and PMS

Our analysis also highlights associations between specific dietary patterns and PMS symptomology. Beyond the BMI interaction, frequent fast-food consumption was independently linked to greater symptom severity. This aligns with evidence that Western-style dietary patterns, characterized by high intake of fast food, soft drinks, and processed meats, are associated with approximately 49% higher odds of PMS [10]. High consumption of calorie-dense, fatty, sugary, and salty foods has been linked to a 3.2-fold increased risk of physical PMS symptoms [14]. Conversely, we observed a nonsignificant trend suggesting a potential protective effect of higher fruit intake, consistent with findings that fruit consumption is associated with reduced behavioral symptoms [14] and that traditional, nutrient-rich diets may mitigate symptom severity.

These findings, derived from a brief FFQ, should be interpreted with caution due to potential measurement error in self-reported dietary habits. Nevertheless, our results underscore diet quality as a key modifiable factor in resource-limited settings. While global evidence regarding diet and PMS remains inconclusive [6,25], this study provides context-specific evidence on the risks of fast-food consumption among Yemeni female students. These exploratory insights suggest that dietary habits form part of a broader lifestyle cluster, warranting further investigation using longitudinal designs or biomarker-confirmed assessments.

Place of residence and contextual stressors

Rural residence was associated with greater PMS severity, a finding consistent with studies from settings where socioeconomic disadvantage correlates with higher symptom burden [1,26]. In our study, we interpret rural residence as a contextual proxy for a cluster of unmeasured adversities, likely including, but not limited to, heightened psychosocial stress, food insecurity, and limited healthcare access. These factors may influence hormonal balance and inflammatory pathways relevant to PMS [3]. This interpretation is supported by research from other conflict-affected populations; for example, studies in the West Bank have documented strong associations between exposure to political violence, human insecurity, and increased PMS symptom reporting [15,22]. The chronic, compounded stressors inherent to Yemen’s protracted conflict likely magnify these environmental and structural risks, informing our interpretation of the rural residence finding.

Physical activity and other lifestyle factors

This study also examined additional lifestyle factors. While evidence from other contexts supports a beneficial role for exercise in managing PMS symptoms [27-29], vigorous physical activity demonstrated only a nonsignificant protective trend in our cohort. The high prevalence of smoking (33.1%) in our sample also warrants attention. Global evidence robustly links smoking with increased risk of PMS, with a meta-analysis reporting an odds ratio of 1.56 and even stronger associations noted for more severe PMDD [30]. This relationship is biologically plausible, as nicotine can interact with hormonal and neurochemical pathways implicated in PMS pathophysiology [30]. In the Yemeni context, tobacco use among university students is a documented public health concern, with usage patterns varying by gender and including both cigarettes and waterpipes [31,32]. Although smoking status did not reach statistical significance for inclusion in the final multivariate model, it remains an important potential confounder or effect modifier. Future studies in similar populations should measure smoking behavior and consumption intensity more precisely to disentangle its relationship with PMS and co-occurring habits such as Qat chewing.

Qat chewing and neurochemical imbalance

A primary novel and contextually significant contribution of this study is the identification of Qat chewing as an independent factor associated with PMS severity. This finding addresses a critical evidence gap, as research on Qat’s effects on menstrual health is notably absent from the literature. The psychoactive alkaloid cathinone is known to affect central monoaminergic systems, including serotonin pathways involved in mood regulation [33], and Qat use has been linked to adverse pregnancy outcomes [34,35]. However, no prior studies have directly examined its relationship with PMS. The observed association is biologically plausible, although likely complex and potentially bidirectional. For instance, severe premenstrual affective symptoms could lead to increased Qat use as a form of self-medication, particularly in high-stress environments. Additionally, Qat use is influenced by socioeconomic and cultural factors that may independently correlate with PMS risk [36-38]. This finding underscores the urgent need for focused investigations into the effects of this culturally embedded practice on women’s reproductive health and highlights the importance of integrating context-specific substance use education into reproductive health strategies.

Implications for practice and policy

The high symptom burden and its associations with modifiable, context-specific factors highlight a critical opportunity for tailored health promotion. The novel findings regarding Qat chewing, along with the synergistic effect of poor diet and overweight status, suggest that public health strategies for young women in conflict-affected settings should move beyond generic guidance. Specifically, integrated interventions addressing nutrition, weight management, and psychoactive substance use are warranted. Feasible, low-cost approaches could include reproductive health education programs that incorporate culturally sensitive messaging on Qat use, nutrition, and stress management within existing university health services and youth programs. Particular attention should be given to rural students, who may face compounded barriers. These findings are primarily hypothesis-generating, and any interventions should be coupled with rigorous evaluation to establish effectiveness.

Study limitations

The findings of this study should be interpreted in light of several methodological limitations. First, the cross-sectional design prevents causal inference; observed associations may reflect how PMS symptoms influence lifestyle choices rather than the reverse. Second, convenience sampling, while necessary in this humanitarian context, limits representativeness and may introduce selection bias, affecting the generalizability of prevalence estimates. Third, reliance on self-reported data poses potential measurement error. Self-reported height and weight may underestimate BMI, while retrospective reporting of symptoms and diet is subject to recall bias. Social desirability may also lead to underreporting of sensitive behaviors such as Qat use. Fourth, the measurement instruments have inherent limitations: the brief FFQ cannot capture detailed nutrient intake or portion sizes, and PMS classification relied on retrospective recall rather than prospective daily charting. Fifth, important contextual confounders, including chronic stress levels, sleep quality, and detailed socioeconomic status, were not quantitatively assessed, despite their likely influence on both lifestyle and PMS risk in a conflict setting. Finally, although the sample size was sufficient for regression analyses, it lacked power for precise prevalence estimation and subgroup analyses (e.g., participants with obesity). Importantly, despite excluding self-reported psychiatric diagnoses, the overlap between PMS symptoms and conditions such as anxiety or depression leaves room for residual confounding by unmeasured mental health issues, representing a major limitation.

## Conclusions

This study demonstrates a high burden of PMS symptoms among female university students in a resource-limited, conflict-affected setting. The exploratory prevalence of moderate-to-severe PMS was substantial, with psychological symptoms constituting the most severe domain. Our analysis identified a cluster of modifiable and context-specific factors, such as overweight status, rural residence, high fast-food consumption, and Qat chewing, associated with greater PMS severity. While the cross-sectional design precludes causal inference, the strength and context-specificity of these associations are noteworthy. In a humanitarian context where specialized gynecological care is often inaccessible, these findings highlight key correlates and underscore a potential opportunity for nonpharmacological public health interventions. Integrating education on nutrition, weight management, and the health impacts of Qat use into existing youth services may represent a feasible strategy to mitigate a significant source of morbidity.

Future longitudinal and intervention-based research is needed to confirm these associations, establish directionality, and evaluate the effectiveness of tailored lifestyle programs. Investigating modifiable correlates of PMS is an essential step toward safeguarding the well-being, academic performance, and resilience of young women navigating the compounded challenges of conflict and recovery.
